# 

**Published:** 1903-11-14

**Authors:** 


JUST OUT. With 3 coloured Plates, and 54 other Illustrations. Price 6/- net.

### HANDBOOK OF DISEASES OF THE EAR

For Students and Practitioners.

By RICHARD LAKE, F.R.C.S. Eng.,
Surgeon to the Royal Ear Hospital.

London: BAILLIÈRE, TINDALL & COX,
8 Henrietta Street, Covent Garden.

### CLINICAL LECTURES ON NEURASTHENIA.

By THOMAS D. SAVILL, M.D.Lond. Second Edition.

"Deals very exhaustively with the subject, and will be read with great interest."—*The Lancet.*

"It should be in the hands of every practitioner who wishes to keep himself well informed on this important subject."—*Treatment.*

H. J. GLAISHER, 57 Wigmore Street, W. 5/- net; 5/4 by post.

JUST ISSUED. EIGHTH EDITION. Crown 8vo. 10s. 6d.

### PHARMACY, MATERIA MEDICA AND THERAPEUTICS

Is now rewritten and printed in large type, and is made a companion to the following volume. It contains an account of the action of **every drug in the B.P.**, as well as a section upon all the newest drugs, with an Introduction to the Art of Dispensing. With woodcuts, prescriptions, &c.

Also FOURTH EDITION. 1055 pages, 8vo. 16s.

### DICTIONARY OF TREATMENT.

Containing a Revised and up-to-date Article upon every Diseased Condition, whether Medical, Surgical, Gynæcological, or Ophthalmological, as well as Articles upon Poisoning, Drowning, and the various Surgical Emergencies and Accidents, arranged for Rapid Reference.

By W. WHITLA, M.A., M.D., Professor of Materia Medica, Queen's College, Belfast; Senior Physician to the Royal Victoria Hospital, &c.

The Publisher will supply both volumes, carriage paid, for 21/-

London: HENRY RENSHAW, 356 Strand.

FOURTH EDITION. Plates contain 93 Coloured and Stereoscopic Figures, 209 Illustrations in the Text, 17/6 net, including Stereoscope.

By P. WATSON WILLIAMS, M.D.(Lond.),
Physician in Charge of Throat Department,
Bristol Royal Infirmary, &c., &c.

### DISEASES OF THE NOSE, PHARYNX, AND LARYNX.

WITH A SECTION ON THE EXAMINATION OF

### THE EAR.

**Press Notices.**—"We know of no better practical guide."—*Practitioner.* "Certain to receive the recommendation of all teachers."—*Brit. Med. Journ.* "We can strongly recommend the book."—*Lancet.* "Excellent......The features of the work—completeness, clear description, and practical utility—are absolutely maintained in the new edition.—(Transn.)." *Internat. Centr f. Laryngologie.*

Bristol: J. WRIGHT & CO. London: SIMPKIN & CO.

### WORKS BY Mr. McADAM ECCLES, M.S., F.R.C.S.,

Assistant Surgeon St. Bartholomew's Hospital, &c.

### HERNIA.

SECOND EDITION, 1902. Price 7/6 net. Illustrated by Numerous Photographs.

### THE IMPERFECTLY DESCENDED TESTIS.

Just published. Profusely Illustrated. Price 7/6.
London: BAILLIÈRE, TINDALL & COX.

Crown 8vo. Illustrated. Price **2s. 6d.** net.

### ANÆSTHETICS. By J. BLUMFELD,

M.D.Cantab., Senior Anæsthetist to St. George's Hospital; Anæsthetist to the Grosvenor Hospital for Women and Children, London, and to the National Dental and Metropolitan Hospitals.

*Lancet.*—"....In writing this handbook the author has been quite successful." *Australasian Medical Gazette.*—"We can strongly commend this book." *Medical Chronicle.*—"The book is one which we can heartily recommend to senior students and practitioners."

London: BAILLIÈRE, TINDALL & COX, 8 Henrietta St., Covent Garden.

### NOTICE TO CORRESPONDENTS.

All MSS., Letters, Books for Review, and other matters intended for the Editor should be addressed The Editor, "The Hospital," The Hospital Buildings, 28 & 29 Southampton Street, Strand, W.C.

The Editor cannot undertake to return rejected MSS., even when accompanied by stamped directed envelope.

### Notice to Advertisers and Subscribers.

All Advertisements, Orders for Copies of The Hospital, and Business Communications should be addressed to **THE MANAGER (Not to the Editor)**, The Hospital, 28 & 29 Southampton Street, Strand, London, W.C.

The Cost of Subscription, post free, is as follows:—Per week, 3½d.; per quarter, 3s. 9d.; per half-year, 7s. 6d.; per annum, 15s. Foreign and Colonial:—Per annum, 19s. 6d., payable, in all cases, in advance.

**NOTICE.—Vol. XXXIV. of "The Hospital" (April 1903 to September 1903), in handsome cloth binding, will shortly be on sale at the office of this Journal, price 7s. 6d. Cases for binding the Numbers contained in this Volume 1s. 6d. Index to Vol. XXXIV., 2½d., post free.**

**The Monthly part for November is now ready. Price 1s., by post, 1s. 3d.**

### BOOKS RECEIVED.

John Murray.

"Treasure and Heart." By Mary Deane.

Grant Richards.

"The Ladies of the Manor." By G. B. Burgen.

W. F. Clay.

"Text-book of Midwifery." By R. Jardine.

J. Wright and Co.

"Pelvic Diagnosis." By E. Stanmore Bishop.

"Medical Examination for Life Assurance." By F. De Havilland Hall.

### Scraps and Gleanings.

It is reported that some suspicious cases with choleraic symptoms have occurred in Bethlehem. There have been eight cases since October 13th, with five deaths. The town is surrounded by a military cordon.

Messrs. Wilson and Stockall, the well-known ambulance manufacturers of Bury, Lancashire, who have been awarded a gold medal for their exhibit at the International Fire Exhibition at Earl's Court, have just received important orders for ambulances from his Highness Prince Alexander of Oldenburg for Russia, and the municipalities of Pretoria and Shanghai.

### The Student of Medicine.

### Appointments.

**Kenneth Anderson**, M.B.Lond., M.R.C.S.Eng., Medical Officer for the Banwell District of the Axbridge Union. **E. E. Argles**, M.R.C.S., L.R.C.P., Assistant Medical Officer St. Marylebone Infirmary. **James Buchanan**, M.B., B.Ch.R.U.I., Medical Officer of the Workhouse of the Watford Union. **W. F. Byford**, M.R.C.S., L.R.C.P.Lond., Medical Officer to his Majesty's Prison at Ruthin. **W. V. Chalmers-Francis**, M.R.C.S., L.R.C.P.Lond., District Medical Officer of the Louth Union. **E. T. Crouch**, M.R.C.S.Eng., L.S.A., District and Workhouse Medical Officer of the Parish of Alverstoke. **H. J. Curtis**, B.S., M.D.Lond., F.R.C.S. Eng., Resident Surgeon to the Memorial Hospital, Bulawayo. **A. Gertrude Grogan**, M.B., B.A.R.U.I., Second Assistant to the Cambridgeshire, etc., Asylum, Wilbourne. **Captain E. M. Illington**, M.R.C.S., L.R.C.P., I.M.S., Clinical Assistant Samaritan Free Hospital, Marylebone Road. **E. B. Leech**, M.B.Camb., M.R.C.S., L.R.C.P., Medical Registrar of the Royal Infirmary, Manchester **J. H. Power**, M.R.C.S., L.R.C.P.Lond., District Medical Officer of the Spalding Union. **L. T. Price**, M.B., Ch.B.Edin., Assistant to the Professor of Anatomy at the University of Edinburgh. **Charles Stennett Redmond**, L.R.C.P.I. and L.R.C.S.I., District Obstetric Physician for the Hulme District of the Manchester Southern and Maternity Hospital. **A. B. Timms**, L.R.C.P. and S.Edin., Assistant House Surgeon to the Cardiff Infirmary. **W. G. Willoughby**, M.D.Lond., D.P.H.Camb., Medical Officer to the Easbourne Education Authority.

### "The Hospital" Institutional Library.

"Hospitals and Asylums of the World." 4 Vols., with a Portfolio of Plans, complete £12 12s.
"Burdett's Hospitals and Charities, 1903." 5s. net.
"Burdett's Official Nursing Directory, 1903." 3s. net.
"Cottage Hospitals, General, Fever, and Convalescent." 10s. 6d.
"Uniform System of Accounts for Hospitals and Public Institutions." New Edition. 4s. net.
"Hospital Expenditure: The Commissariat." 2s. 6d.
"A Legal Handbook for the Use of Hospital Authorities." 2s. 6d.
"The Cottage Hospital Case Book or Register of Patients." 10s. and 12s. 6d.

All these are published by the Scientific Press, Ltd., and may be obtained through any bookseller, or direct from the publishers, 28 and 29 Southampton Street, Strand, London, W.C.

### Medical Appointments.

MEDICAL DEPARTMENT OF THE NAVY.
Admiralty, S.W., September 17th, 1903.
An EXAMINATION of CANDIDATES for entry into the Medical Department of the Royal Navy will be held on the 16th NOVEMBER next and following days at Examination Hall, Thames Embankment.
The forms to be filled up by candidates will be supplied on application to this Department.
H. F. NORBURY, Director-General.
(1481)

WOLVERHAMPTON UNION.
ASSISTANT MEDICAL OFFICER (RESIDENT).

The Guardians of the Poor of the Wolverhampton Union invite applications for the appointment of ASSISTANT MEDICAL OFFICER at their Workhouse (recently erected and equipped), Heath Town, Wolverhampton.
Candidates must be single or widowers without children dependent, and must be duly qualified and registered. The appointment will be subject to the approval of the Local Government Board, and the provisions of the Poor Law Officers' Superannuation Act, 1896.
Salary £130 per annum, together with Apartments, Rations and Washing.
Information as to the duties may be obtained from the Clerk to the Guardians, Union Offices, Wolverhampton, to whom applications must be forwarded (upon forms which he will supply on request) not later than TUESDAY, December 1st, 1903.
Canvassing the Guardians will be deemed a disqualification.
By order,
FRANK HARRISON,
Clerk to the Guardians. (1912)

### THE HANDBOOK OF THE OPEN-AIR TREATMENT

By Dr. CHARLES REINHARDT

(Visiting Physician to Hailey Sanatorium, Wallingford, and Stourfield Park Sanatorium, Bournemouth),

Contains Views, Descriptions, and Information respecting 30 Sanatoria in Great Britain.

Price: Cloth, 2/6; Paper, 1/- (postage 4d.)

The "HEALTH RESORT" Publishing Office, 379 Strand, London, W.C.

Printed by SPOTTISWOODE & CO. LTD., New Street Square, E.C.; and Published by the Proprietors, THE SCIENTIFIC PRESS, LIMITED, at 28 & 29 Southampton Street, Strand, London, W.C.

To be had of all Booksellers and Newsagents in the United Kingdom, and at all the Railway Bookstalls of Messrs. W. H Smith & Son.

### To Nurses.

DEAR NURSES,—We have learnt by observation that something larger and simpler is needed in the nursing of private cases than can be found amongst the ordinary case books published. Therefore most nurses buy an exercise book, and arrange each one themselves. Now you need not waste your valuable time in ruling and writing. Our Case Book is ready for use for the price of an exercise book. It is much neater and clearer than anything you can arrange for yourselves. If you like our book, please show it to others. They also will be glad to render their reports in concise form. It helps the doctor, and is appreciated by him.

Yours faithfully,
THE PUBLISHERS.

### A NEW CASE BOOK FOR PRIVATE NURSING.

**The Size of an Exercise Book.**

**($7\frac{1}{2}$ in. by $9\frac{1}{2}$ in.).**

**Arranged for Day and Night Nurse's Report, with rulings in accordance with what has been found in practice to be the most suitable and approved System for Pay Hospitals, Nursing Homes, Private Nurses, &c.**

Price
**3d. net,**
Post free $4\frac{1}{2}$d.

DAY NURSE'S REPORT.

*Date*.............

| Time | Nourishment | Quantity | Urine | Stimulants, Medicines, and Remarks |
|---|---|---|---|---|
| | | | | |

| Total Nourishment | Bowels | Summary |
|---|---|---|
| | | |

(Facsimile of ruling.)

***Obtainable of all Booksellers.***

**Special Quotations for a Quantity can be procured from the Publishers,**

The SCIENTIFIC PRESS, LTD.,
28 & 29 Southampton Street, Strand, London, W.C.

### WORKS FOR NURSES.

**LESSONS ON MASSAGE.** By Mrs. MARGARET D. PALMER, Manager of the Massage Department and Instructor of Massage to the Nursing Staff of the London Hospital. Second Edition enlarged and revised; pp. xvi. + 262 with 118 Illustrations, plain and coloured. *Just Published.* Price 7/6 net.

**A MANUAL FOR STUDENTS OF MASSAGE.** By Miss M. A. ELLISON, L.O.S. With 50 original Illustrations. Price 3/6 net.

**A MANUAL FOR HOME NURSING.** By BERNARD MYERS, M.D., M.R.C.S., Surgeon and Lecturer to St. John's Ambulance Association. *Just Published.* Price 2/6 net.

**NOTES ON HOME NURSING.** By Miss D. M. GOLDIE, Lecturer on Nursing, Hygiene, &c., to the County Councils of London, Surrey and Middlesex. *Just Published.* Price 1/6 net.

**THE FEEDING AND HYGIENE OF INFANTS AND CHILDREN.** By JOHN McCAW, M.D., M.R.C.P., Physician to the Belfast Hospital for Children. *Just Published.* Price 2/6.

**DISEASES OF THE HAIR, INCLUDING GREYNESS, BALDNESS, &c., AND THEIR TREATMENT.** By DAVID WALSH, M.D., Western Hospital for Diseases of the Skin. Price 2/6 net.

**MENSTRUATION AND ITS DISORDERS.** By ARTHUR E. GILES M.D., B.Sc., F.R.C.S., Surgeon Chelsea Hospital for Women. Price 2/6 net.

**MOVABLE ATLAS OF THE ANATOMY AND PHYSIOLOGY OF THE FEMALE GENERATIVE ORGANS AND OF PREGNANCY.** Second Edition. With Text. By ARTHUR E. GILES, M.D., F.R.C.S. Price 3/- net.

**MOVABLE ATLAS OF THE ANATOMY AND PHYSIOLOGY OF THE CHILD.** With Explanatory Text. By D'ARCY POWER, M.A.Oxon, M.B., F.R.C.S. Price 3/- net.

**BAILLIÈRE'S POPULAR MOVABLE ATLAS OF THE ANATOMY AND PHYSIOLOGY OF MAN.** Size 16 by 7½ inches. With Explanatory Text. By W. S. FURNEAUX, Special Science Teacher London School Board. Price 3/6 net.

London: BAILLIÈRE, TINDALL & COX, 8 Henrietta Street, Covent Garden.

### NOW READY,

A REPRINT OF

### A NURSING HANDBOOK

TOGETHER WITH S. MAW, SON AND SONS'

### COMPLETE CATALOGUE OF NURSING REQUISITES.

The above work is now republished at a cost of 1s. per copy. Messrs. S. M., S. and S. have been compelled to make this charge on account of the extreme popularity of the work and the great expense incurred in its production.

***Post Free on receipt of 1s.***

Nurses' Bags.

### S. MAW, SON and SONS,

7 to 12 ALDERSGATE STREET, LONDON, E.C.

